# Genome-wide identification and expression analysis of AP2/ERF transcription factors in sugarcane (*Saccharum spontaneum* L.)

**DOI:** 10.1186/s12864-020-07076-x

**Published:** 2020-10-02

**Authors:** Peiting Li, Zhe Chai, Pingping Lin, Chaohua Huang, Guoqiang Huang, Liangnian Xu, Zuhu Deng, Muqing Zhang, Yu Zhang, Xinwang Zhao

**Affiliations:** 1 National Engineering Research Center for Sugarcane & Guangxi Key Laboratory for Sugarcane Biology, Fujian Agriculture and Forestry University, Fuzhou, 350002 China; 2 State Key Laboratory for Conservation and Utilization of Subtropical Agro-Biological Resources & Guangxi Key Laboratory for Sugarcane Biology, Guangxi University, Nanning, 530005 China; 3 Fujian Provincial Key Laboratory of Plant Functional Biology, College of Life Sciences, Fujian Agriculture and Forestry University, Fuzhou, 350002 China; 4 Key Laboratory of Sugarcane Biology and Genetic Breeding Ministry of Agriculture, Fujian Agriculture and Forestry University, Fuzhou, 350002 China

**Keywords:** Sugarcane, Transcription factor, *AP2/ERF* gene, Abiotic stress

## Abstract

**Background:**

APETALA2/ETHYLENE RESPONSIVE FACTOR (AP2/ERF) transcription factors play essential roles in plant growth, development, metabolism, and responses to biotic and abiotic stresses. However, few studies concerning *AP2/ERF* genes in sugarcane which are the most critical sugar and energy crops worldwide.

**Results:**

A total of 218 *AP2/ERF* genes were identified in the *Saccharum spontaneum* genome. Phylogenetic analysis showed that these genes could be divided into four groups, including 43 *AP2s,* 160 *ERFs* and *Dehydration-responsive element-binding* (*DREB*) factors, 11 *ABI3/VPs (RAV),* and four *Soloist* genes. These genes were unevenly distributed on 32 chromosomes. The structural analysis of *SsAP2/ERF* genes showed that 91 *SsAP2/ERFs* lacked introns. Sugarcane and sorghum had a collinear relationship between 168 *SsAP2/ERF* genes and sorghum *AP2/ERF* genes that reflected their similarity. Multiple cis-regulatory elements (CREs) present in the *SsAP2/ERF* promoter were related to abiotic stresses, suggesting that SsAP2/ERF activity could contribute to sugarcane adaptation to environmental changes. The tissue-specific analysis showed spatiotemporal expression of *SsAP2/ERF* in the stems and leaves of sugarcane at different development stages. In ten sugarcane samples, 39 *SsAP2/ERFs* were not expressed, whereas 58 *SsAP2/ERFs* were expressed in all samples. Quantitative PCR experiments showed that *SsERF52* expression was up-regulated under salt stress, but suppressed under dehydration stress. *SsSoloist4* had the most considerable upregulation in response to treatment with the exogenous hormones ABA and GA. Within 3 h of ABA or PEG6000 treatment, *SsSoloist4* expression was up-regulated, indicating that this gene could play a role in the responses to ABA and GA-associated dehydration stress. Analysis of *AP2/ERF* gene expression patterns under different treatments indicated that *SsAP2/ERF* genes played an essential role in dehydration and salt stress responses of *S. spontaneum.*

**Conclusions:**

In this study, a total of 218 members of the AP2 / ERF superfamily were identified in sugarcane, and their genetic structure, evolution characteristics, and expression patterns were studied and analyzed. The results of this study provide a foundation for future analyses to elucidate the importance of AP2/ERF transcription factors in the function and molecular breeding of sugarcane.

## Background

Unfavorable environmental conditions have severe impacts on crop yields, and thus strategies to improve crop survival under adverse conditions are needed [1]. Plants respond to environmental stresses via complex regulatory mechanisms that elicit a series of physiological and biochemical responses [2]. Transcription factors play an essential role in converting stress-induced signals into cellular responses. When various abiotic stresses stimulate plants, signaling pathways involving molecules such as abscisic acid and ethylene are activated [3]. This activation is often associated with changes in the expression of transcription factors, which specifically bind to trans-acting elements in promoter regions at downstream target genes. For cis-acting elements, the regulatory effect is executed through the activation or inhibition of downstream functional genes [4]. In plants, these two main processes are involved in responses to biotic or abiotic stress, which are mediated by various transcription factors. The AP2/EREBP (APETALA2/ethylene response element-binding protein) superfamily comprises a large class of transcription factors in plants. Multiple studies have demonstrated that AP2/ERF transcription factors in plants are essential for stresses responses, and their expression is regulated by plant hormones [5, 6]. In response to stresses in plants, AP2/ERF transcription factors’ expression is regulated to coordinate growth under stresses [7].

AP2/ERF family members contain the highly conserved AP2/ERF DNA binding domain. Based on sequence similarities and the number of AP2/ERF domains, the AP2/ERF superfamily can be divided into four categories: AP2, ERF (Ethylene-responsive factor), RAV (related to ABI3/VP1), and Soloist [8]. In most cases, the AP2 family contains proteins with two AP2/ERF domains involved in regulating plant developmental processes [9]. RAV proteins contain two different DNA-binding domains (AP2 and B3), which are regulated by ethylene or brassinosteroid hormones and also are involved in biotic and abiotic stress responses [10, 11]. The ERF family is divided into two subfamilies: ERF and DREB (dewater-responsive element binding). Both ERF and DREB contain only one AP2/ERF domain and are critical regulators of plant responses to biotic and abiotic stresses [12]. The Soloist group contains only one AP2 conserved domain and forms a separate group based on its significant structural difference from the other AP2/ERF family members. However, there are limited evidence that the Soloist group members are positive regulators of SA-mediated plant defense against pathogens [13].

AP2/ERF transcription factors have well-documented functions in plant growth and development. For example, in *Arabidopsis thaliana, WIND1* (*RAP2.4*, *At1g78080*) is involved in controlling cell dedifferentiation that, in turn, affects proliferation, axillary bud growth, and bud branching [14]. The ERF family gene *OsEATB* in rice limits internode elongation by down-regulating gibberellin biosynthesis genes [15]. In tomatoes [16], grapes [17], Chinese jujube [18], and bananas [19], some AP2/ERF superfamily members are involved in fruit maturation.

AP2/ERF transcription factors also play a crucial role in abiotic stress responses in plants. For example, *OsEREBP1* and *OsEREBP2* modulate the expression of *OsRMC*, a negative regulator of rice salt stress [20]. Overexpression of maize *ZmDBP3* enhances tolerance of transgenic *Arabidopsis* to drought and cold stresses [21]. In contrast, overexpression of the *WIN1* gene from Sorghum confers drought resistance to *Arabidopsis* by regulating the epidermis [22]. The ERF and DREB families, in particular, contain members that have excellent performance in response to abiotic stress. The DREB protein can specifically bind the A/GCCGAC (DRE/CRT) element related to genes involved in responses to ABA, drought, and low temperature, while ERF subfamily members can interact with the core sequence AGCCGCC (GCC-box) of the ethylene response element (ERE). Such binding to ERE element regulates responses to ethylene and abiotic stresses, and promotes disease resistance [23]. However, many reports suggest that both *Arabidopsis* ERF and DREB can be combined with DRE/CRT or ERE elements, indicating that they have a potential role in abiotic [24]. DREBs belong to the ABA-independent signal transduction pathway and are divided into two subclasses: DREB1/CBF and DREB2. *DREB1/CBF* genes are thought to be involved mainly in low-temperature sensation, whereas most *DREB2* genes participate in response to water or heat shock stress [25]. However, there is increasing evidence that the stress regulation mediated by the *DREB1/CBF* and *DREB2* genes is species-specific [26]. AP2/ERF transcription factors are also likely to be essential mediators of plant resistance, but few studies concern these genes’ activity in sugarcane.

Sugarcane (*Saccharum spp*.) is the most important crop for sugar and biofuel [27]. Sugarcane provides 75 and 40% of global sugar and ethanol production [27, 28]. Damage to sugarcane caused by environmental stresses thus can have substantial economic impacts [29]. Drought stress during sugarcane growth reduces productivity by between 30 and 70%, and minimizes sucrose formation and sucrose recovery by 5% [30]. Genome-wide analysis of the presence of AP2/ERF transcription factors in wild sugarcane *Saccharum spontaneum* species would be necessary for sugarcane resistance research. In this study, we identified members of the *AP2/ERF* superfamily in the *S. spontaneum* genome. We also carried out the phylogenetic relationships, gene structure, conserved domains, promoters, chromosomal location distribution, and gene duplication. The effects of *AP2/ERF* genes on sugarcane adaptation to environmental stresses were analyzed to enhance our understanding of the mechanisms.

## Results

### Identification and classification of AP2/ERF genes

A total of 218 complete *AP2/ERF* genes were identified in the sugarcane genome database. The identified genes ranged from 416 to 22,786 bp and were predicted to encode proteins with 127–876 amino acids (aa) (Additional files 1 and 2). Based on sequence similarities and the number of conserved AP2 domains (Additional files 3 and 4), the *AP2/ERF* genes were divided into four families: AP2, ERF, RAV, and Soloist. The AP2 family had 43 genes, of which 36 had two identical conserved AP2 domains, and 7 had only one conserved AP2 domain (*SsAP2–37* to *SsAP2–43*). The ERF family had 160 genes, 59 and 101 were assigned to DREB (*SsDREB1* to *SsDREB59*) and ERF (*SsERF1* to *SsERF101*) subfamily. The RAV family comprised of 11 members (*SsRAV1 to SsRAV11*) with conserved AP2 and B3 domains. Another four genes (*SsSoloist1* to *SsSoloist4*) with one or two conserved AP2 domain had less similarity to ERF or AP2. Instead, these four genes had higher homology to *Arabidopsis At4g13040*, and thus were classified into the Soloist. Because no reliable naming method was defined in previous research on the AP2/ERF family, our naming convention was based on the grouping of 218 sequences and their chromosomal positions. The total number of AP2/ERF superfamily candidate genes in sugarcane was higher than that in the *Arabidopsis* (147) and rice (181) [8]. However, the number of ERF family members was similar but slightly higher (122 and 145 for *Arabidopsis* and rice, respectively).

### Phylogenetic analysis

**Fig. 1:**
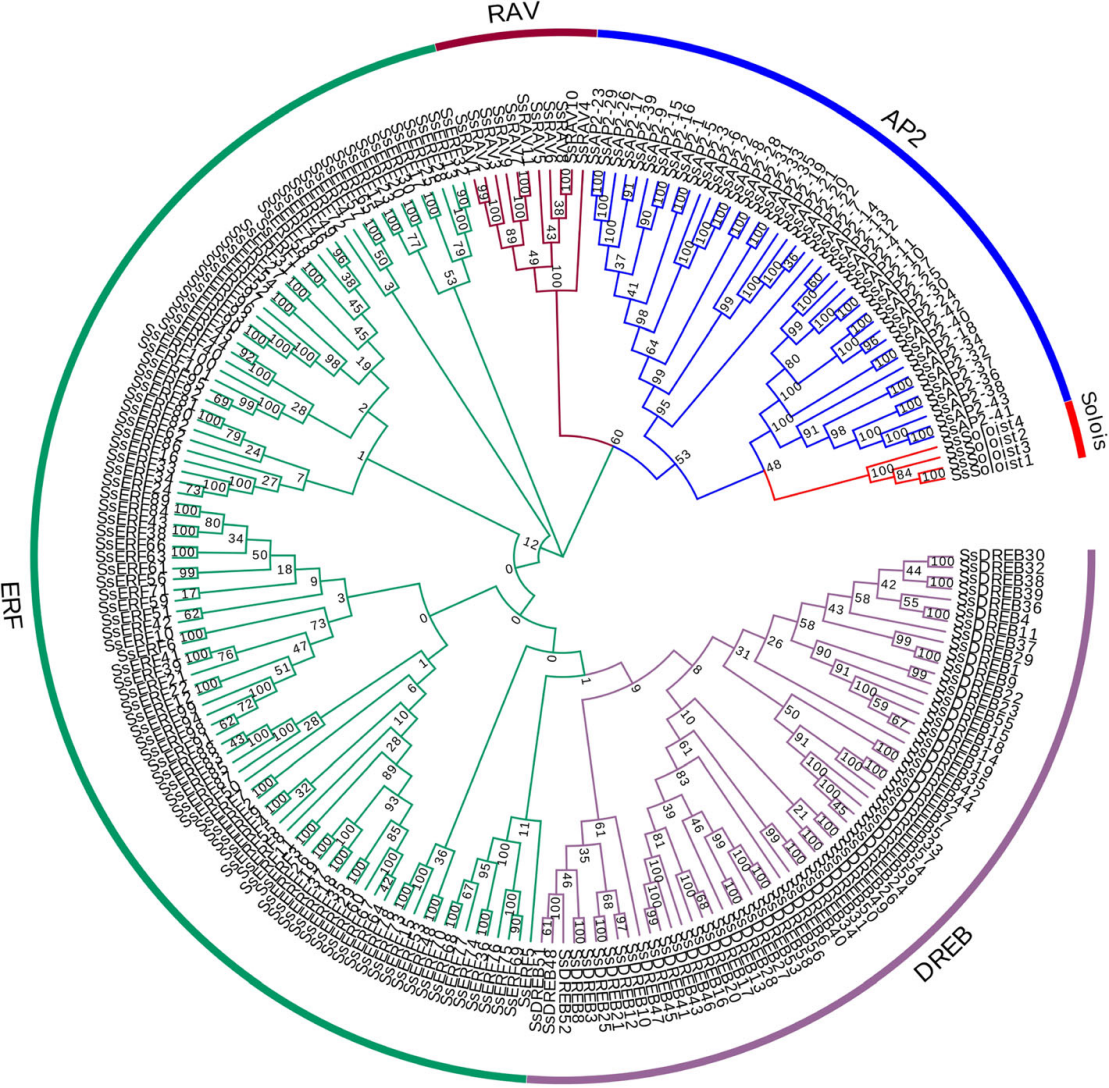
Phylogenetic tree of *AP2/ERF* genes in sugarcane. ERF, DREB, AP2, RAV, Soloist families were presented in different colors

**Fig. 2:**
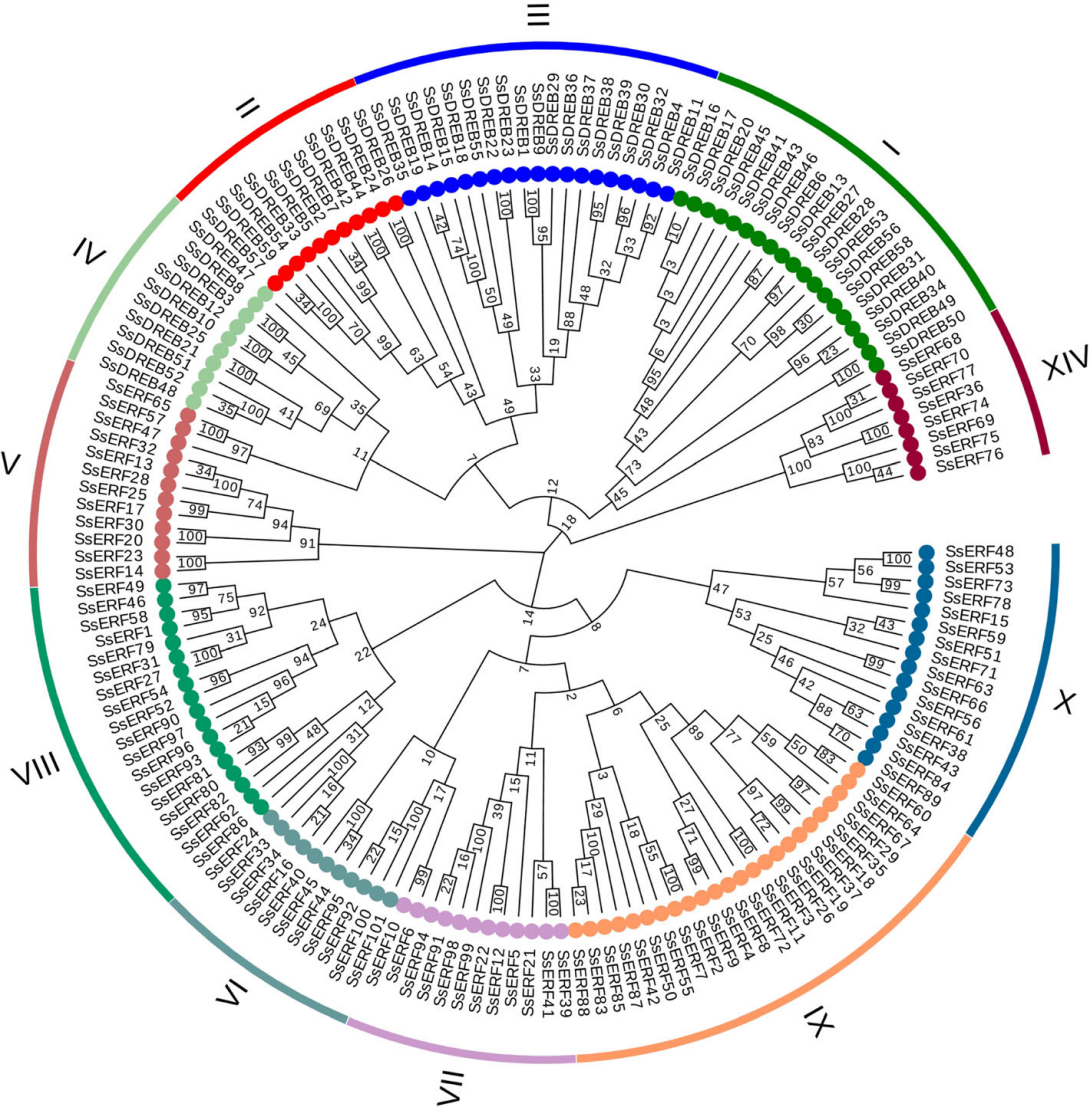
Phylogenetic tree of *ERF* and *DREB* subfamily genes in sugarcane. Each group has a different color. The 11 clades (I-XIV) of the *ERF* and *DREB* subfamily genes were divided according to previous classifications in Arabidopsis and rice

To analyze the evolutionary relationship of the *SsAP2/ERF* genes, we constructed phylogenetic trees based on the amino acid sequences encoded by these genes (Fig. 1). The phylogenetic tree clustered all of the *SsAP2/ERF* genes into four main branches: AP2, ERF, RAV, and Soloist. According to the classification criteria in *Arabidopsis* and rice [8], the ERF family was divided into two subfamilies: the DREB subfamily (59 members, divided into groups I, II, III, and IV, containing 19, 11, 19, and 10 members, respectively) and the ERF subfamily (101 members divided into groups V, VI, VII, VIII, IX, X, and XIV containing 12, 11, 12, 18, 24, 16 and 8 members, respectively) (Fig. 2). Notably, most species lacked a XIV group, although *Os08g41030* in rice had a conserved domain similar to these eight sugarcane members and could be classified into the XIV group. *Arabidopsis* had no genes that were consistent with the XIV group. Thus, an additional examination is needed to determine whether functional differentiation occurred and its significance.

### Gene structure and conserved motif analysis

**Fig. 3:**
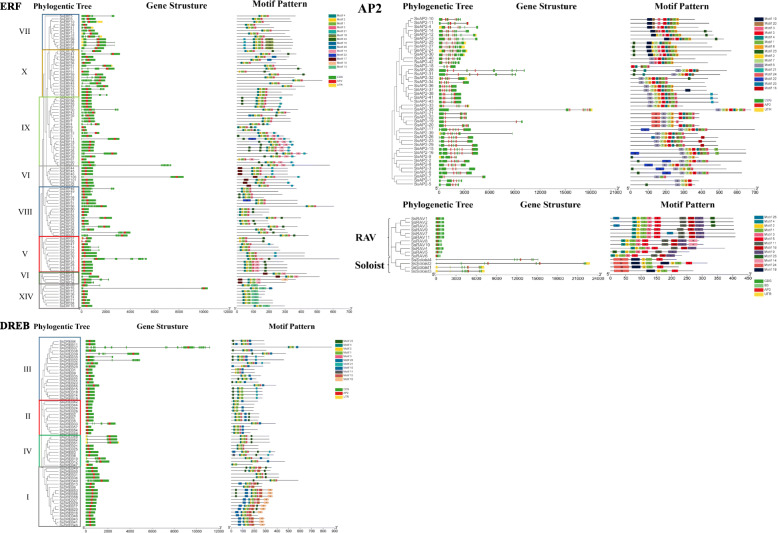
Phylogenetic relationships, gene structure, and architecture of conserved protein motifs in *AP2/ERF* genes from sugarcane. The phylogenetic tree was constructed based on the full-length sequences of sugarcane AP2/ERF proteins using MEGA7 software—exon and intron structure of sugarcane AP2/ERF genes. Yellow boxes indicated untranslated 5 - and 3 -regions; green boxes indicated exons; gray lines indicated introns. The AP2 domains were highlighted by red boxes and the B3 domain by light green boxes. The motifs, numbers 1–25, were displayed in different colored boxes. The sequence information for each motif was provided in Additional file 6. The protein and gene length can be estimated using the scale at the bottom

To characterize the structural diversity of SsAP2/ERF genes, we analyzed the number of introns and exons and the distribution of conserved domains in the coding sequence of a single SsAP2/ERF gene (Additional file 5: Figure S6). Through gene structure analysis, differences during the AP2, ERF, and RAV families could be observed, and the results from the conserved domain analysis were consistent with those of previous studies. The number of introns among the different family genes varied markedly (Fig. 3), and 42% of AP2/ERF genes had no intron. Most of DREB genes had no intron except for 18 genes, such as *SsDREB16*, *SsDREB20*, *SsDREB49*, *SsDREB50*, which the number of introns ranged from 1 to 24. A total of 40 ERF genes had no intron. Unlike the ERF genes, 42 AP2 genes had the number of introns ranging from 2 to 9. For the 11 RAV genes, *SsRAV9* had one intron, *SsRAV5* had six introns, while the remaining nine RAV genes had on intron. The number of introns of the four Soloist genes was between 6 and 9. Interestingly, the genes clustered into the same branch on the phylogenetic tree had similar exon-intron structure. For example, DREB group II genes had no intron except the gene *SsDREB33,* which had only one intron.

We used MEME to investigate *SsAP2/ERF* gene diversity further and predicted the presence of 25 conserved motifs in the AP2/ERF family (Additional file 6 and Figure 3). Motif-1 and -2 were present in all SsAP2/ERF protein sequences; Both motif-3 and -4 were available in most members of the AP2, ERF, DREB, and RAV subfamily; Motif-6, − 7, − 8, − 20, and − 22 were found only in the AP2 family; Motif-10, − 12 and − 15 appeared in group I; Motif-13 appeared in group XIV, and Motif-9 and -11 were unique to the RAV family. We also found that Motif-1, − 2, and − 7 existed in the AP2 conserved domain, whereas Motif-9, − 11, and − 16 were located in the B3 domain (Fig. 3). As expected, most close relatives among subfamily members had similar motif compositions, suggesting that AP2/ERF proteins within the same subfamily were functionally identical.

### Chromosome distribution

**Fig. 4:**
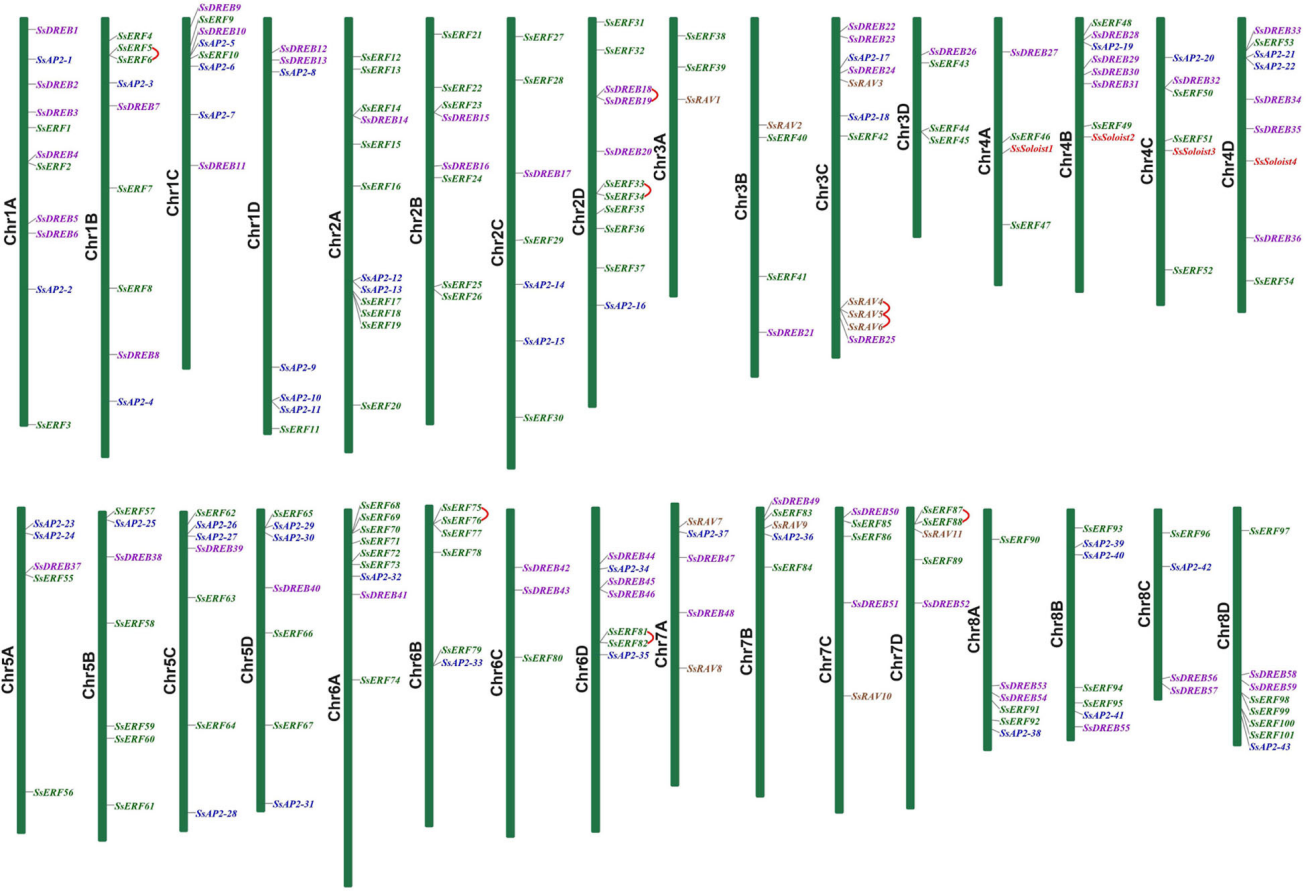
Schematic representations for the chromosomal distribution of sugarcane *AP2/ERF* genes. A red line between the two gene names indicated that they were tandem repeat gene pairs. Different colors distinguish family genes names of *AP2, ERF, DREB, RAV, and Soloist.* The chromosome number was indicated to the left of each chromosome

The 218 identified *SsAP2/ERF* genes were mapped to 32 chromosomes, and the distribution across the chromosomes varied widely (Fig. 4). Chr2A had the most genes (12), whereas Chr6C and Chr8C had only three genes (Additional file 7). At least one of the 59 *SsDREB* and 101 *SsERF* genes could be found on each of the 32 chromosomes, and the four *Soloist* genes were distributed on four homologous chromosomes, Chr4A, Chr4B, Chr4C, and Chr4D. Members of the *SsRAV* family were present only on chromosomes 3 and 7. Surprisingly, 50% of the *SsAP2/ERF* genes localized at one of the four rearrangement chromosomes (SsChr02, SsChr05, SsChr06 and SsChr07) and nine *SsAP2/ERF* genes were present in the rearranged regions, including SsChr5 (Sb05S) 57.6–89.1 Mbp, SsChr6 (Sb05L) 54.6–90.6 Mbp, SsChr7 (Sb08S) 62.0–83.3 Mbp, SsChr2 (Sb08L) 98.5–125.9 Mbp [31].

### Gene duplication analysis

**Fig. 5:**
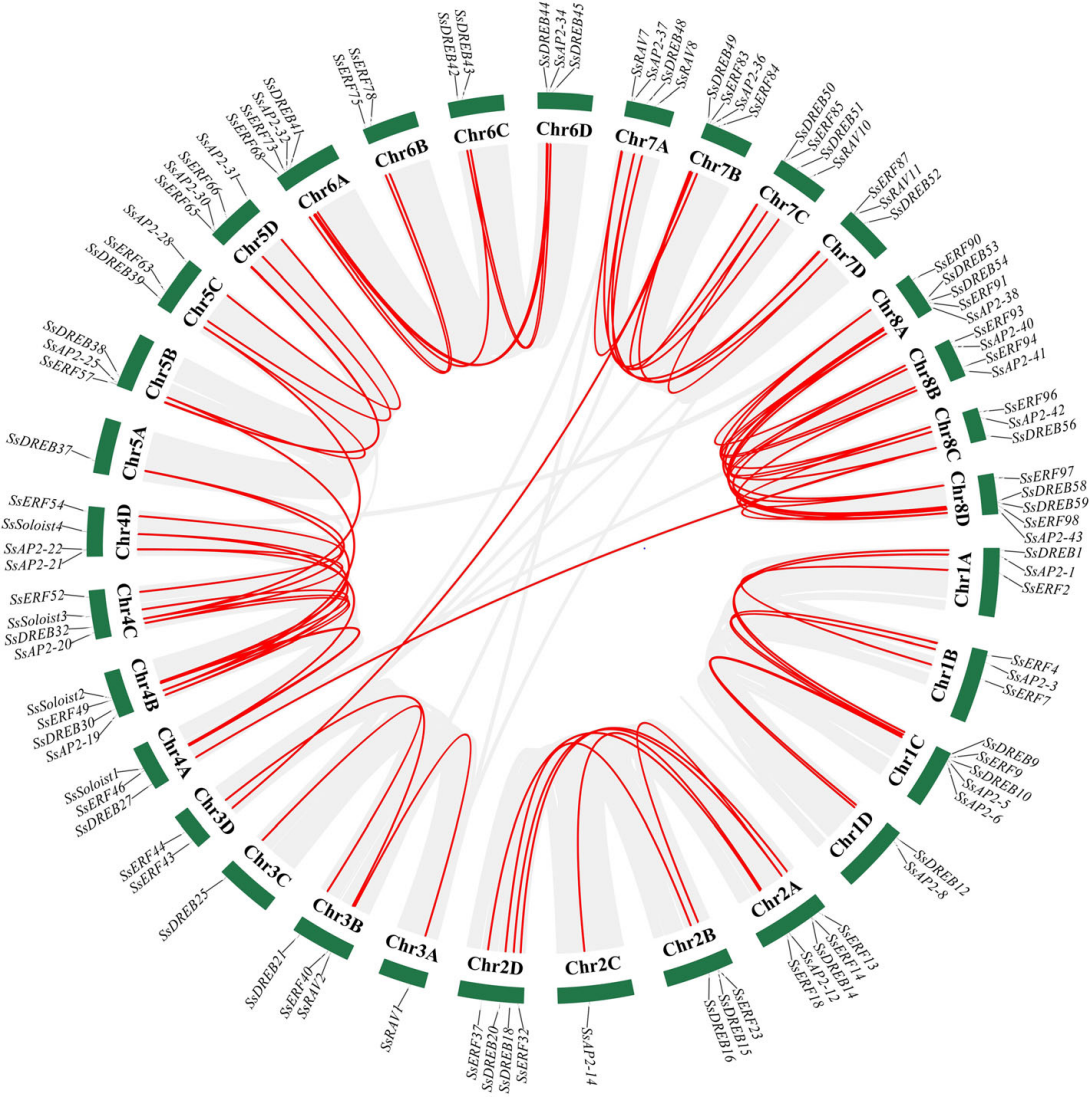
Schematic representations for the interchromosomal relationships of sugarcane *AP2/ERF* genes. Gray lines indicated all syntenic blocks in the sugarcane genome, and the red lines indicated duplicated *AP2/ERF* gene pairs

Multiple studies indicated that gene families evolved due to genome-wide duplication, segmental duplication or tandem duplication, and gene diversification occurred after these duplication events [32]. To explore *SsAP2/ERF* genes evolution, we studied tandem and segmental duplication events of these genes using chromosomal information for *S. spontaneum* (Additional file 8). A total of eight pairs of tandem duplication genes were detected, of which two pairs were *RAV* genes, and six ones were *ERF* genes (Fig. 4). Three genes, *SsRAV4*, *SsRAV5,* and *SsRAV6,* were two tandem duplication gene pairs located on the same chromosome and adjacent to each other. Besides, 70 pairs of 103 *SsAP2/ERF* segmental duplication genes were detected (Fig. 5). Among these, 53 occurred between alleles, and 17 were between non-alleles. There were four pairs of *SsAP2/ERF* segmental duplication gene pairs distributed on Chr4 and Chr5. The distribution might be due to the segments of both Chr4 and Chr5 chromosomes from the ancestral A4 chromosome [31].

The time for the divergence of the *SsAP2/ERF* genes was estimated to have undergone tandem and segmental duplication based on Ks values (Additional file 8). The divergence time for the eight *SsAP2/ERF* tandem duplication pairs ranged from 14.2 to 104.2 million years ago (mya), illustrating that these *SsAP2/ERF*s arose from recent gene duplication events in *S. spontaneum*. Sixty-four segmental duplication pairs appeared earlier, based on a divergence time that ranged from 4.9 to 89.9 mya, whereas the other four segmental duplication pairs were ancient, diverging between 164.7 and 212.1 mya.

We calculated Ka/Ks values for *SsAP2/ERF* genes in tandem and segmental duplications to detect which selection type promoted *AP2/ERF* gene family evolution (Additional file 8). The Ka/Ks ratio of tandem duplication gene pairs among *AP2/ERF* genes ranged from 0.17 to 1.24, with an average of 0.57. Tandem duplication gene pairs having a Ka/Ks ratio < 1 accounted for 89% of the genes tested. The Ka/Ks ratio for segmental duplication gene pairs ranged from 0 to 2.6, with an average of 0.62, and 92% of pairs had Ka/Ks < 1. These results indicated that repetitive *SsAP2/ERF* genes were under intense purification selection pressure, and the duplication-producing gene had enormously evolved and maintained its functional stability. Meanwhile, nine replicate gene pairs had Ka/Ks > 1, indicating that they were subject to positive selection after duplication differentiation.

### Evolutionary analysis of *SsAP2/ERFs* and other plant *AP2/ERFs*

**Fig. 6:**
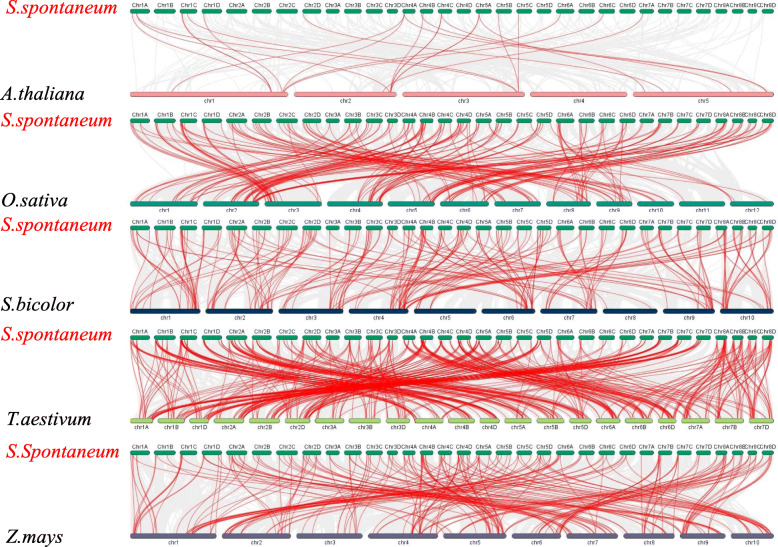
Synteny analysis of *AP2/ERF* genes between sugarcane and five representative plant species. Gray lines in the background indicate the collinear blocks within sugarcane and other plant genomes, while the red lines highlighted the syntenic AP2/ERF gene pairs. The specie names with the prefixes ‘*S. spontaneum*’, ‘*A. thaliana*’, ‘*O. sativa*’, ‘*S. bicolor*’, ‘*T. aestivum*’ and ‘*Z. mays*’ indicated *Saccharum spontaneum*, *Arabidopsis thaliana*, *Oryza sativa*, *Sorghum bicolor*, *Triticum aestivum* and *Zea mays*, respectively

A syntenic map of five representative species was constructed to examine the evolutionary origin of the *AP2/ERF* genes in sugarcane (Fig. 6 and Additional file 9). A total of 168 *SsAP2/ERF* genes were synonymous with genes in Sorghum, followed by rice (151), wheat (145), maize (143), and *Arabidopsis* (19). Orthologous gene pairs were distributed across all chromosomes of *S. spontaneum*. Some *SsAP2/ERF* genes were associated with at least three pairs of corresponding genes (particularly *AP2/ERF* genes in sugarcane and wheat), suggesting that these genes played an essential role in *SsAP2/ERF* superfamily evolution. Interestingly, some collinear gene pairs (90 *SsAP2/ERF* genes) were available in monocotyledonous plants (sugarcane and rice/sorghum/wheat/maize), but absent in the dicotyledonous ones (sugarcane and *Arabidopsis*).

### Analysis of putative cis-regulatory elements (CREs)

**Fig. 7:**
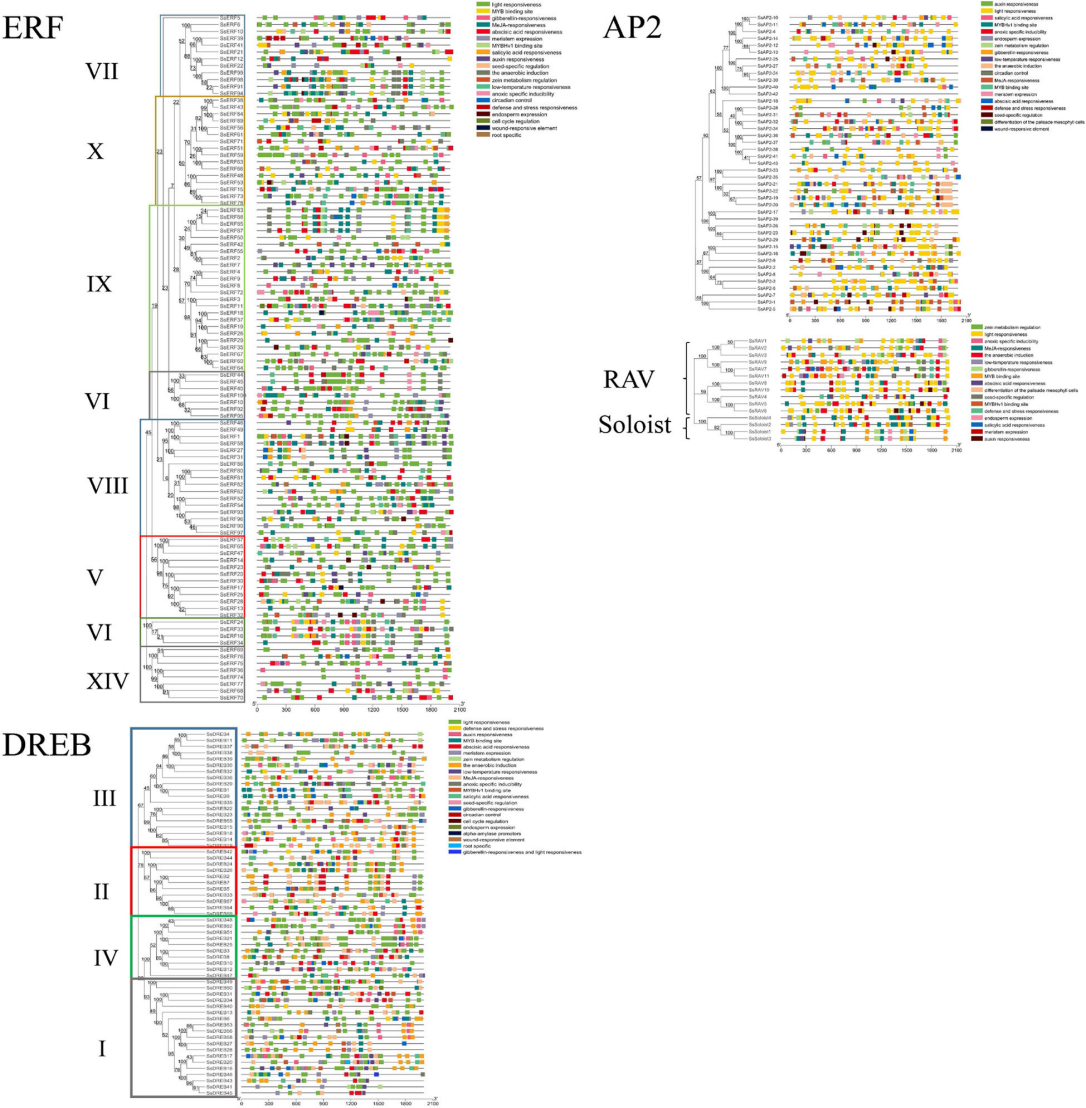
Cis-acting elements and phylogenetic trees in the promoter region of the sugarcane AP2/ERF genes. The 2000 bp promoter region upstream of the gene was analyzed. Different colored boxes represent different cis-acting elements

CRE is a non-coding DNA sequence that regulates transcription in a defined temporal/spatial expression pattern. CREs are essential for understanding expression differences and mutations. We used PlantCare to identify putative CREs of 2000 bp located on SsAP2/ERF genes (Fig. 7 and Additional file 10). These CREs were classified according to their function, and the number of CRE in each sequence was determined. Many CREs responded to different hormone inducers, such as abscisic acid (ABA), salicylic acid (SA), methyl jasmonate (MeJA), gibberellin (GA), and auxin. Among them, ABA and MeJA response elements were the most common. There are also CREs genes related to various stress stimuli (such as a wound, defense, and stress, anoxic, anaerobic, and low-temperature). A total of 182 SsAP2/ERF genes contained the abscisic acid response element, 126 genes with the gibberellin response element, and 192 genes with the MeJA response element. Another 108 SsAP2 / ERF genes contained low-temperature response elements, while 155 genes had light responsiveness elements. Each family had ten types of CREs, including light responsiveness, endosperm expression, anoxic specific inducibility, auxin, gibberellin, abscisic acid, MeJA, and salicylic acid responsiveness, MYBHv1, and MYB binding site. DREB genes had the most CREs species, among which alpha-amylase promoters and root-specific CREs only existed in DREB subfamily genes. The CREs of Soloist family genes were the least, mainly hormone and stress response. The five families of genes had similar CREs types, but the number of copies of each CRE was different. In some *AP2/ERF* genes, the promoter region contained cis-acting elements for various transcription factors, such as MYB, MYC, ERE and DRE elements.

### Expression pattern of *AP2/ERF* genes during sugarcane development

To understand specific spatiotemporal expression patterns of *SsAP2/ERF* genes, we analyzed the identified genes’ expression profiles in ten different tissues and at different developmental stages using publicly available gene expression data (Additional files 11 and 12). Among the *SsAP2/ERF* genes examined, 39 were not expressed in all tissue samples, 58 were expressed in all ten tissue types (FPKM > 0), and 27 were constitutively expressed (FPKM > 2). Total 19 genes in DREB group I was all expressed in at least one sample, among which *SsDREB17*, *SsDREB27,* and *SsDREB28* were highly expressed in all ones, indicating that these group I members were likely essential functional genes. The expression levels of 26 *SsAP2/ERF* genes increased during leaf maturation, suggesting that they might play an important role in leaf growth and development. The expression of *SsDREB38*, *SsERF91*, *SsERF98*, *SsERF39*, and *SsERF99* in stems was much higher than that in leaves in the three developmental stages. Among them, four *ERF* genes belonged to ERF group VII, indicating that they played an essential role in cane stems. Expressions of 11 genes, including *SsDREB56*, *SsDREB48*, *SsERF15,* and *SsRAV3*, *SsSoloist1,* and *SsSoloist2*, were higher in the three stages of leaf development than those in the stems, indicating that these genes played an important role in leaf development. The expression of ten genes, including *SsDREB55*, *SsERF62,* and *SsERF54* in different tissues in the pre-mature and mature stages, was higher than that seen in various tissues in the seedling stage. Among these genes, many were in the ERF subfamily, indicating that they played an essential role in the mature sugarcane stage.

### Expression analyses of *SsAP2/ERF* genes in response to abiotic stress and hormone treatments

**Fig. 8:**
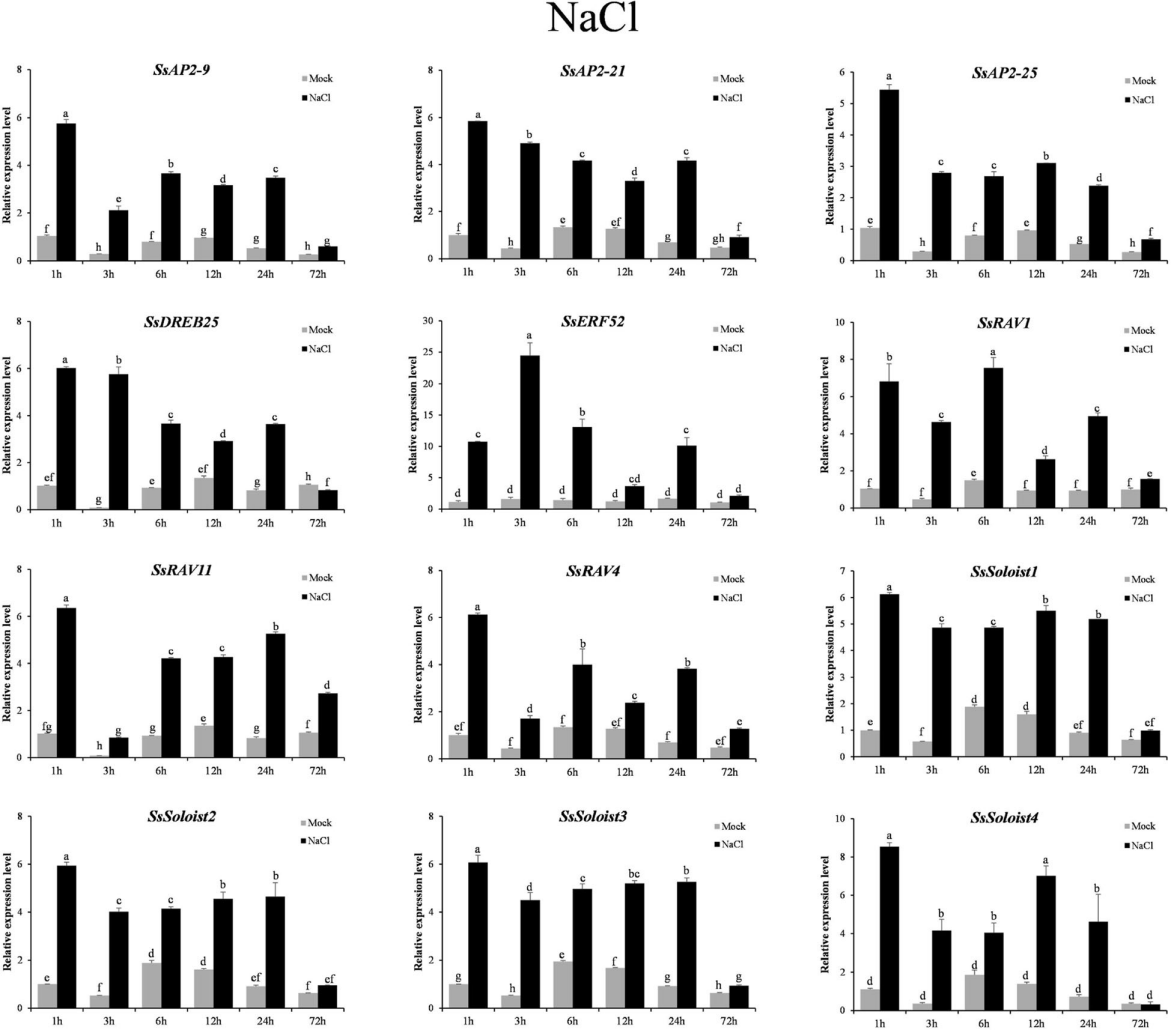
Expression profiles of 12 selected SsAP2/ERF genes in response to NaCl stress treatments. *The 25 s-RNA* gene was used as the internal control and to normalize expression data. Relative transcript abundance was normalized relative to S mock (1-h untreated control group) treatment. Error bars represented the standard deviation of the mean. Different lowercase letters indicated significant differences among mean values (one-way ANOVA with Ducan’s multiple range test; *P* < 0.05). The results were based on three replicates in three independent experiments

To further confirm whether *SsAP2/ERF* gene expression was affected by different abiotic stresses and hormone treatments, we examined 12 genes from five families. We analyzed their expression patterns following different treatments (Fig. 8 and Additional file 13). NaCl, PEG6000, and ABA treatment could induce the expression of all 12 genes. After 3 h of treatment, compared with the control group simultaneously, the up-regulation multiple was the highest among all treatment time points. Different treatments had varying degrees of influence on gene expression. Compared with the control group, all the genes were induced by NaCl, except that *SsSoloist4* down-regulated expression at 72 h of NaCl treatment. The up-regulation multiple was significantly higher than the other three treatments. Some genes had opposite expression patterns under different treatments. For example, *SsERF52* was down-regulated at 1, 6, and 12 h after PEG6000 treatment. Although *SsERF52* was up-regulated 2.15 times at 3 h, its up-regulation was significantly lower than that of other genes at 3 h. Meanwhile, the expression of *SsERF52* was induced by salt stress at six treatment times. The up-regulated multiple was 15.21 times at 3 h, which was significantly higher than other genes, indicating that the response of *SsERF 52* to salt stress and dehydration stress was different. The other four genes, *SsAP2–25*, *SsDREB25*, *SsRAV11,* and *SsRAV4*, had no significant up-down multiple changes at all time points after GA treatment but were induced by the other three treatments.

## Discussion

As one of the largest gene families in plants, the AP2/ERF family plays a vital role in multiple physiological and biochemical processes by regulating the expression of genes that participate in response to various stress conditions in *Arabidopsis*, rice [8], maize [33], poplar [34], and grapes [17]. Still, there is limited information concerning the regulation and structure of these genes in sugarcane. In this study, we examined whole-genome data for the wild sugarcane species *S. spontaneum* to identify genes encoding AP2/ERF family transcription factors, and to analyze their roles based on published expression data for *S. spontaneum* tissue and qRT-PCR results.

Genome-wide analysis of sugarcane identified 218 *SsAP2/ERF* genes, which was higher than that in rice (164) [8], wheat (117) [35], and *Arabidopsis* (145) [8], but fewer than that for maize (292) [33]. The genome size varies in different plants, rice (466 Mb), wheat (4.94 Gb), *Arabidopsis* (125 Mb), maize (2.3 Gb), and *S. spontaneum* (3.36 Gb), indicating that the number of AP2/ERF superfamily members is relatively stable and does not have an absolute correlation with genome size. However, since AP85–441 (1n = 4x = 32) used to sequence the *S. spontaneum* genome was haploid and produced from the culture of the octoploid SES208, the number of *AP2/ERF* genes in octoploid *S. spontaneum* could be over 218 [31]. Gene duplication plays a vital role in gene family amplification to generates gene clusters or hotspots via tandem repetitive and segmental duplication to produce homologous genes that expand the total number of genes. Segmental duplication events found in 104 *SsAP2/ERF* genes also validated this possibility. *S. spontaneum* has undergone two Whole Genome Duplication (WGD) events overtime in which its homologous chromosomes underwent duplication from one to two and then to four [31]. *SsAP2/ERF* gene repeats likely occurred during these two WGDs. We also found evidence that purification selection was the main driving force behind the *SsAP2/ERF* gene family’s evolution. By evaluating the gene structure of *AP2/ERF* TF, all *SsAP2* and *SsSoloist* genes had introns, whereas 50.6 and 83.3% of *SsERF/DREB* and *SsRAV* genes, respectively, had no introns. Loss of introns in genes after segmental duplication occurred more rapidly than intron acquisition [36]. Also, some studies showed that the number and distribution of introns were related to plant evolution [37], such that introns might have been lost from ERF and RAV family genes during the evolution of higher plants. From these results, ERF and RAV gene differentiation might occur later in *S. spontaneum* evolution.

Syntenic analysis of *AP2/ERF* was carried out to discover the evolutionary relationship of the *SsAP2/ERF* genes in five monocotyledonous plants (*O. sativa, S. bicolor*, *T. aestivum*, *Z. mays*, *S. spontaneum*) and one dicotyledonous plant (*A. thaliana*). In this analysis, *SsAP2/ERF* genes had higher homology with *AP2/ERF* genes from the four kinds of grass and less homology with *Arabidopsis*. More homologous genes between sugarcane and wheat are likely due to the larger genome size and gene number in wheat [38]. By analyzing the homology of *SsAP2/ERF* and *S. bicolor AP2/ERF* genes, we found that 168 *SsAP2/ERF* genes likely existed before the number of *S. spontaneum* chromosomes was reduced from 10 to 8. Also, 63–70% of identified *SsAP2/ERF* genes were homologous in sorghum (168), rice (147), maize (139), and wheat (145). This finding indicated that the *AP2/ERF* family in different plants might have evolved from a common ancestor.

Due to the plasticity of the *AP2/ERF* genes and individual family members’ specificity, members of this family were likely to be important targets for genetic engineering and breeding to improve crops [39]. The gene expression patterns are essential for the prediction of gene function prediction. Analysis of tissue- and growth stage-specific expression showed that *SsAP2/ERF*s were widely expressed in sugarcane at the seedling stage and leaves during early maturity and the mature stage and in different stem segments, indicating that these genes played essential roles in sugarcane growth and development. *DREB* family genes in group I was highly expressed in leaves at all development stages and in three stem segments, suggesting that these genes might be involved across the entire development process of sugarcane leaves and stems. The expression levels of 29 *SsAP2/ERF* genes in stems during the seedling stage were lower than those in the early maturity stage and mature stage. Among them, 28 were members of the DREB and ERF family, which might be related to sugar accumulation in the sugarcane stem and consistent with previous studies for other plants. For example, compared with wild rice, transgenic rice carrying the ERF protein *TSRF1* had 30–60% increases in proline and soluble sugar content that could enhance the osmotic tolerance of rice [40].

Earlier studies of *AP2/ERF* family genes in other plants emphasized their role in response to hormonal and abiotic stresses. *OsEREBP1* attenuates disease caused by *Xoo* and confers drought and submergence tolerance in transgenic rice [41]. Induction of the AP2/ERF transcription factor *CRL5* promoted crown root initiation by inhibiting cytokinin signaling [42]. In the present study, the expression patterns of 12 *SsAP2/ERF* genes in response to NaCl, PEG6000, ABA, and GA treatments suggested that these genes had essential roles in abiotic stress and hormone responses sugarcane. qRT-PCR verification revealed that the expressions of these 12 *SsAP2/ERF* genes distributed across the five subfamilies were significantly induced by NaCl treatment, similar to other species’ findings, including the increase in expression of the *AP2/ERF* family gene *CAP2* in chickpeas and soybeans exposed to salt stress [43]. *GmERF7* expression increased salt tolerance in tomato [44], whereas the pepper pathogen-induced transcription factor *RAV*_*1*_ played a vital role in bacterial salt stress tolerance [45]. Here we showed that the expression of *SsERF52* was up-regulated by up to 15-fold after NaCl treatment. This gene had the highest homology with the wheat salt-responsive gene, *TaERF4* [46]. Like *TaERF4* in wheat, *SsERF52* expression was not induced by exogenous abscisic acid (ABA). Overexpression of the *Arabidopsis* AP2/ERF gene *HARDY* improved drought tolerance by reducing the transpiration of transgenic *Trifolium alexandrinum* L [47]. Drought stress induced expression of the DERB *OsAP21* in rice [48], and here we found that expression of 12 *SsAP2/ERF* genes was induced at 3 h after PEG6000 treatment. The expression level of *SsERF52* after PEG6000 treatment was opposite that after NaCl treatment, with inhibition of gene expression at several time points. Multiple lines of evidence indicated that genes expressed during dehydration and salt stress responses in plants partially overlapped. For example, transgenic plants that overexpressed *Arabidopsis OsMYB3R-2* have enhanced cold tolerance, drought tolerance, and salt tolerance [49]. Increasing amounts of evidence showed that *AP2/ERF* genes were regulated by hormones such as ABA and GA, and thus these genes played an essential role in hormone signaling networks [50]. In the present study, promoter analysis indicated that most *SsAP2/ERF* genes’ promoter regions contained multiple cis-acting elements related to ABA responses. qRT-PCR analysis of the 12 selected *SsAP2/ERF* genes showed that, except for *SsERF52*, expressions of these genes were induced by ABA. *AP2 / ERF* genes played an active role in ABA-mediated stress response, a critical hormone that regulated abiotic stress responses (including drought, salinity, cold, and heat stress) [50]. For example, *ANT* [51] in *Arabidopsis* was induced by ABA to up-regulate genes containing DRE and ABRE elements. *ANT* is homologous to *SsAP2–21*, indicating that *SsAP2/ERF* genes might regulate abiotic stress through ABA-dependent pathways. After exogenous GA treatment, the expression levels of *SsRAV4*, *SsRAV11*, *SsDREB25*, and *SsAP2–25* were not affected, but GA induced other genes’ expression. Our research indicated that some *SsAP2/ERF* genes were differentially expressed under various abiotic stresses and hormone treatments compared with the control group, suggesting that this gene family was essential for environmental adaptation. In particular, the expression patterns of some *SsAP2/ERF* genes and their homologs varied. For example, the *SsSoloist4* gene expression was induced to varying degrees by the four stress treatments. These expression patterns differed from those for its *Arabidopsis* homolog *At4g13040*, an active regulator of disease defenses, and induced by SA treatment. However, the expression of this gene was inhibited by low temperature and salt stress. Given the antagonistic roles of SA and ABA in plant defense, *At4g13040* expression may have opposing effects in response to ABA and SA treatment. Compared with the control group, *SsSoloist4* expression was up-regulated after 3 h of ABA treatment, indicating that this gene’s regulation mechanism likely differed from that for *At4g13040* under abiotic stress.

Therefore, it could be seen from the above experiments that 12 *SsAP2 / ERF* genes were induced and expressed by NaCl and PEG6000 in varying degrees. There were many CREs related to ABA response in the promoter region of these genes, such as ABRE, and more than one copy, which meaned that their fast-induced expression on exogenous ABA treatment might also be mediated by themselves. A total of 12 *AP2 / ERF* genes were positively regulated by ABA, which further confirmed the result. Among the 12 AP2 / ERF genes, only *SsAP2–21* had no CRE related to GA response. However, after GA treatment, the expression of 12 AP2 / ERF genes had no significant correlation with the number of TATC-box box, showing that gene expression was a complex biological process, regulated by many factors. The results also showed that the number of CREs were related to abiotic stress. Still, there was no significant correlation between CREs and 12 SsAP2/ERF genes’ gene expression under dehydration and salt stress.

AP2 / ERF gene is regulated by ethylene and brassinosteroid when the plant exposed to salt, low temperature, and flooding stress [52]. AP2 / ERF, especially the ERF subfamily, plays a role as the primary downstream regulator of the ETH signaling pathway [52, 53]. For example, in Arabidopsis, *EIN3* activate *ERF1*, *ESE*, and their downstream stress-related genes, promoting salt tolerance. ETH signal transduction is a necessary condition for plant salt tolerance [54]. However, ethylene’s regulation to stress through AP2 / ERF is very complex, which needs further study. BRs also plays a vital role in the abiotic stress response of plants [55]. Recent studies have shown that *AP2 / ERF* gene is involved in regulating BRs on drought stress and plant growth [56]. BRs signal negatively regulates DREB gene *TINY* through the phosphorylation of *BIN2*, and *TINY* positively regulates drought response and inhibits BRs mediated plant growth through the antagonistic effect of *TINY*-*BES1*. These findings provide useful insight to study the relationship between SsAP2 / ERF and BRs in the future. Plants will inevitably suffer from oxidative stress when subjected to biotic and abiotic stresses [57]. The change of oxidation balance might be complex and hurt plants. AP2 / ERF gene is involved in the regulation of oxidative stress response in plants [58]. Oxidative stress strongly induced the ERF gene *SICRF1* in tomato [59]. Cytokinin response factor 6 (*CRF6*) in Arabidopsis played a negative role in oxidative stress response, indicating that the cytokinin mediated the oxidative stress response regulated by *AP2 / ERF* gene [60]. However, the mechanism of plant response to abiotic stress, including oxidative stress, is still unclear. Sugarcane response to oxidative stress-mediated by *SsAP2 / ERF* genes is needed for further research in the future. Our results could provide a foundation for identifying novel candidate *AP2/ERF* genes as targets for genetic engineering of novel sugarcane germplasm that will produce plants with enhanced tolerance of abiotic stresses.

## Conclusions

This study comprehensively analyzed the supergene family of sugarcane AP2 / ERF. The 218 identified *AP2 / ERF* superfamily genes were classified in detail. Their evolutionary characteristics, expression patterns in different sugarcane tissues and growth stages, and their response to abiotic stress and hormones were studied. These results provided valuable resources to understand better the biological role of the sugarcane *AP2 / ERF* genes.

## Methods

### Identification and classification of members AP2/ERF superfamily genes in sugarcane

The genome sequences and the sequence information of sugarcane were downloaded from the *S. spontaneum* AP85–441 genome (http://www.life.illinois.edu/ming/downloads/Spontaneum_genome/) [31]. The protein sequences of AP2/ERF superfamily genes in rice and *Arabidopsis* were collected from the NCBI (https://www.ncbi.nlm.nih.gov/). These proteins were used as query sequences in the local BLAST program (Basic Local Alignment Search Tool) to find members of AP2/ERF superfamily genes of the sugarcane genome with the following parameters: expected values ≤1E-5. All BLAST hits were checked and searched for conserved AP2 domains online using the Search Pfam feature of the Pfam (https://pfam.xfam.org/) website under default parameters. In addition, the results of Pfam were verified again using the NCBI CDD tool (https://www.ncbi.nlm.nih.gov/Structure/bwrpsb/bwrpsb.cgi/) and the cutoff set to 0.01.

### Phylogenetic analysis of sugarcane *AP2/ERF* genes

Multiple alignments of candidate *AP2/ERF* genes were performed to explore the phylogenetic relationship of sugarcane *AP2/ERF* genes using ClustalW [61] with default parameters. The results were used to construct phylogenetic trees by the neighbor-joining method and were then visualized using MEGA 6.0 software [62]. The phylogenetic trees were generated using complete protein sequences with the following parameters: pair-wise deletion, Poisson correction, and 1000 bootstrap replicate.

### Gene structure and conserved motif analyses

Conserved motifs of AP2/ERF proteins were identified using the online tool Multiple Em for Motif Elicitation (MEME) version 5.0.5 [63] (http://meme-suite.org/tools/meme/) with the following parameters: (1) the number of occurrences of a single motif distributed among the sequences within the model was set to zero or one per sequence; (2) the maximum number of motifs found was set as 25; (3) the optimum motif width was set to ≥6 and ≤ 50; and (4) motifs with a matched E-value should be below 0.05. Gene structure was investigated using GSDS 2.0 (http://gsds.cbi.pku.edu.cn/) [64]. We used TBtools software [65] to integrate phylogenetic trees, conserved motifs, and gene structure results.

### Chromosomal distribution and duplication analysis of AP2/ERF superfamily genes

The chromosomal distribution information of the identified genes was searched against the reference sugarcane genome database, and the results obtained were visualized using TBtools software. Analysis of gene Duplication events using Multiple collinear scanning toolkits (MCScanX) [66]. The syntenic relationship between the *SsAP2/ERF* genes and *AP2/ERF* genes from selected plants was determined by using Dual Synteny Plotter software (https://github.com/CJ-Chen/TBtools/). The putative duplication events were detected for the *AP2/ERF* genes. Tandem duplication was identified as two proteins with a greater than 40% similarity and separated by four or fewer gene loci; others were identified as segmental duplications, separated by more than five genes. Non-synonymous (ka) and synonymous (ks) substitution of each duplicated AP2/ERF genes were calculated using KaKs_Calculator 2.0 [67]. The divergence time (T) was calculated by T = Ks/ (2× 6.1 × 10^− 9^) × 10^− 6^ Mya [68]. These results were visualized using TBtools.

### Analysis of cis-acting elements of AP2/ERF superfamily genes

Two thousand bp upstream of the transcriptional start site of each *SsAP2/ERF* gene was selected to inspect potential CREs. They were submitted to the PlantCARE website (http://bioinformatics.psb.ugent.be/webtools/plantcare/html/).

### Transcriptome data source and bioinformatic analysis

Sugarcane tissue-specific expression data included leaves and stems at the seedling stage of 35 days and the early maturity stage of 9 months and the maturity stage of 12 months (Additional file 14). All SsAP2 / ERF FPKMs (Segmentals Per Kilobase of transcript per Million segmental mapped) was used to make heat maps and cluster analysis through TBtools.

### Plant materials

Sugarcane variety ROC22 is from Guangxi University, Guangxi, China. All the materials were cultured in Murashige and Skoog’s medium containing 3% sucrose and 0.7% agar until seedling stage [69]. The seedlings were then transplanted to the greenhouse with light intensity of 100 μ mol / m-2S-1, light cycle of 16:8-h (light / dark), temperature of 28 °C, and treated with hormone at 5–7 leaf stage and simulated abiotic stresses. Hormonal treatment was sprayed with abscisic acid (ABA, 100 μM) and gibberellin (GA, 100 μM). Abiotic stress treatment was irrigated with NaCl (250 mM) to simulate salt stress, and PEG6000 (20%) was used to simulate dehydration stress. All the young leaves were taken for samples with the time points are 1, 3, 6, 12, 24, and 72 h. The collected samples were quickly placed in liquid nitrogen and stored in a − 80 °C refrigerator for subsequent RNA extraction.

### Quantitative real-time PCR (qRT-PCR) analysis

Each qRT-PCR reaction mixture (20 μL) comprised of 10 μL Taq polymerase (SYBR Premix Ex TaqII; Takara), 2 μL of each forward and reverse primers (2 μM), 2 μL of cDNA, and 6 μL of water. The RNA expression level was normalized to the level of 25S-rRNA expression. The amplification was conducted in LightCycler 96 Real-Time PCR System (Roche Lightcyler® 480). A standard thermal profile for SYBR Premix was as followed: cDNA synthesis at 37 °C for 15 min and enzyme inactivation at 85 °C for 5 s. qPCR conditions were: initial denaturation 95 °C for 30s, denaturation 95 °C for 5 s, annealing and extension 60 °C for 30s. Transcripts expression levels were calculated with the 2^−ΔΔCt^ method, as previously mentioned in Livak and Schmittgen [70]. Primers used for this analysis are mentioned in Additional file 15.

### Data statistics

All data were analyzed for variance using IBM SPSS Statistics. Statistical methods were used to compare the significance levels of LSD (least significant difference test, LSD) at 0.05.

## Supplementary information

**Additional file 1 **List of the 218 *SsAP2/ERF* genes identified in this study.

**Additional file 2 **Sequence analysis of 218 *SsAP2 / ERF* genes.

**Additional file 3.** Conserved domains of AP2/ERF superfamily proteins identified by HMMSCAN.

**Additional file 4.** Multiple alignments of deduced amino acid sequences of the AP2/ERF and B3 DNA-binding domains of AP2/ERF superfamily proteins.

**Additional file 5 **Gene structures of *AP2/ERF* superfamily genes. The exon/intron structure was visualized by the Gene Structure Display Server 2.0 (Visualized). The data of gene structure was based on the gene annotation model of *S. spontaneum* L. genome.

**Additional file 6 **Summary of motifs in *AP2/ERF* superfamily genes by MEME analysis.

**Additional file 7 **Chromosome distribution of *AP2/ERF* superfamily genes in the *Saccharum spontaneum* L. genome.

**Additional file 8 **Segmentally and tandemly duplicated *SsAP2/ERF* gene pairs.

**Additional file 9.** One-to-one orthologous relationships between sugarcane and other five plant species.

**Additional file 10 **List of cis-acting elements in the *AP2 / ERF* superfamily genes’ promoter region.

**Additional file 11 **RNA-seq data of 218 *SsAP2/ERF* genes that were used in this study.

**Additional file 12.** Tissue-specific expression profile of AP2 / ERF gene in sugarcane.

**Additional file 13 **Expression profiles of 12 selected *SsAP2/ERF* genes in response to various abiotic stress treatments and hormone treatments.

**Additional file 14.** Sugarcane tissue-specific expression data.

**Additional file 15.** Sequences of the primers used in this study.

## Data Availability

All data generated or analyzed during this study are included in this published article and its supplementary information files (from Additional file 1 to Additional file 15). The genome sequences and the sequence information of sugarcane were downloaded from the *S. spontaneum* AP85–441 genome (http://www.life.illinois.edu/ming/downloads/Spontaneum_genome/).

## Abbreviations

*S. spontaneum*: *Saccharum spontaneum* L
*SsAP2/ERF*: Sugarcane AP2/ERF
SsChr: *Saccharum spontaneum* chromosome
FPKM: Segmentals Per Kilobase of transcript per Million segmental mapped
A4: Ancestral chromosome A4
ABA: Abscisic acid
GA: Gibberellins
PEG: Polyethylene Glycol
SA: Salicylic acid
*Xoo*: *Xanthomonas oryzae pv. Oryzae*
ETH: Ethylene
BRs: Brassinosteroids
