# Supplementary material for: Genome-wide identification and expression analysis of AP2/ERF transcription factors in sugarcane (Saccharum spontaneum L.)
Source: BMC Genomics. 2020 Oct 2;21:685. doi: 10.1186/s12864-020-07076-x (PMC7531145; doi:10.1186/s12864-020-07076-x)
Supplement: Supplementary file 4 — Additional file 4. Multiple alignments of deduced amino acid sequences of the AP2/ERF and B3 DNA-binding domains of AP2/ERF superfamily proteins. [file 12864_2020_7076_MOESM4_ESM.docx]

**Additional file 4** Multiple alignments of deduced amino acid sequences of the AP2/ERF and B3 DNA-binding domains of AP2/ERF superfamily proteins.

**Fig. S1**. Multiple alignments of DREB subfamily proteins using clustal method by Bioedit software. The conserved Val-16 (V) and Glu-21 (E) residues were important for distinguish of DREB family genes. Although several genes did not contain these two amino acid residues, they showed a close relationship by phylogenetic analysis and thereby were classified into DREB family.

**Fig. S2**. Multiple alignments of ERF subfamily proteins using clustal method by Bioedit software. The conserved Ala-15 (A) and Asp-20(D) residues were important for distinguish of ERF family genes.

A

B

**Fig. S3**. Multiple alignments of AP2 subfamily proteins using clustal method by Bioedit software.

A Fig S2. Multiple alignments of AP2 subfamily proteins using clustal method by Bioedit software.

B

**Fig. S4**. Multiple alignments of RAV subfamily proteins using clustal method by Bioedit software.

**Fig. S5**. Multiple alignments of Soloist subfamily proteins using clustal method by Bioedit software.
