# Supplementary material for: Genome-wide identification and expression analysis of AP2/ERF transcription factors in sugarcane (Saccharum spontaneum L.)
Source: BMC Genomics. 2020 Oct 2;21:685. doi: 10.1186/s12864-020-07076-x (PMC7531145; doi:10.1186/s12864-020-07076-x)
Supplement: Supplementary file 5 — Additional file 5 Gene structures of AP2/ERF superfamily genes. The exon/intron structure was visualized by the Gene Structure Display Server 2.0 (Visualized). The data of gene structure was based on the gene annotation model of S. spontaneum L. genome. [file 12864_2020_7076_MOESM5_ESM.docx]

**Additional file 5** Gene structures of *AP2/ERF* superfamily genes.


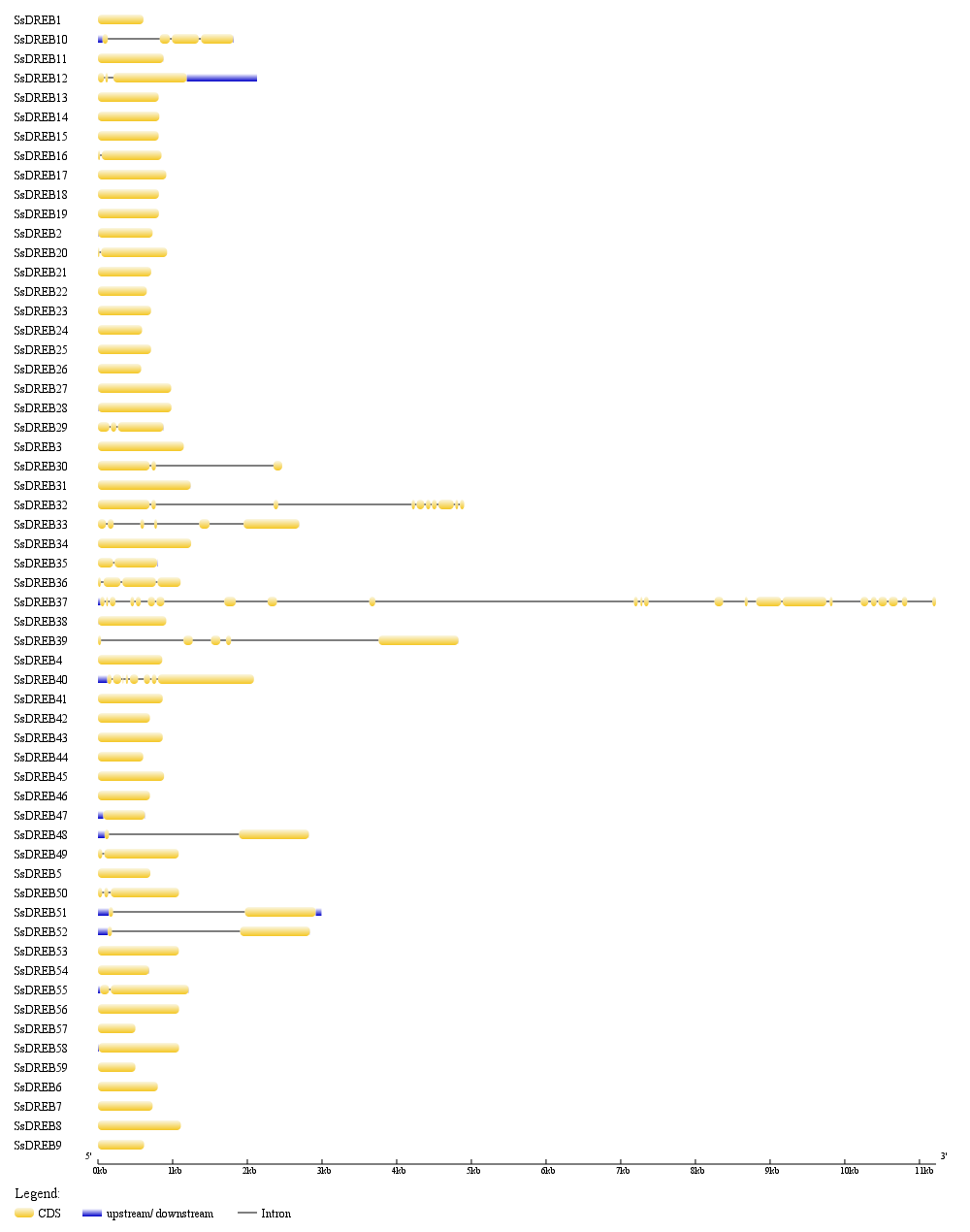


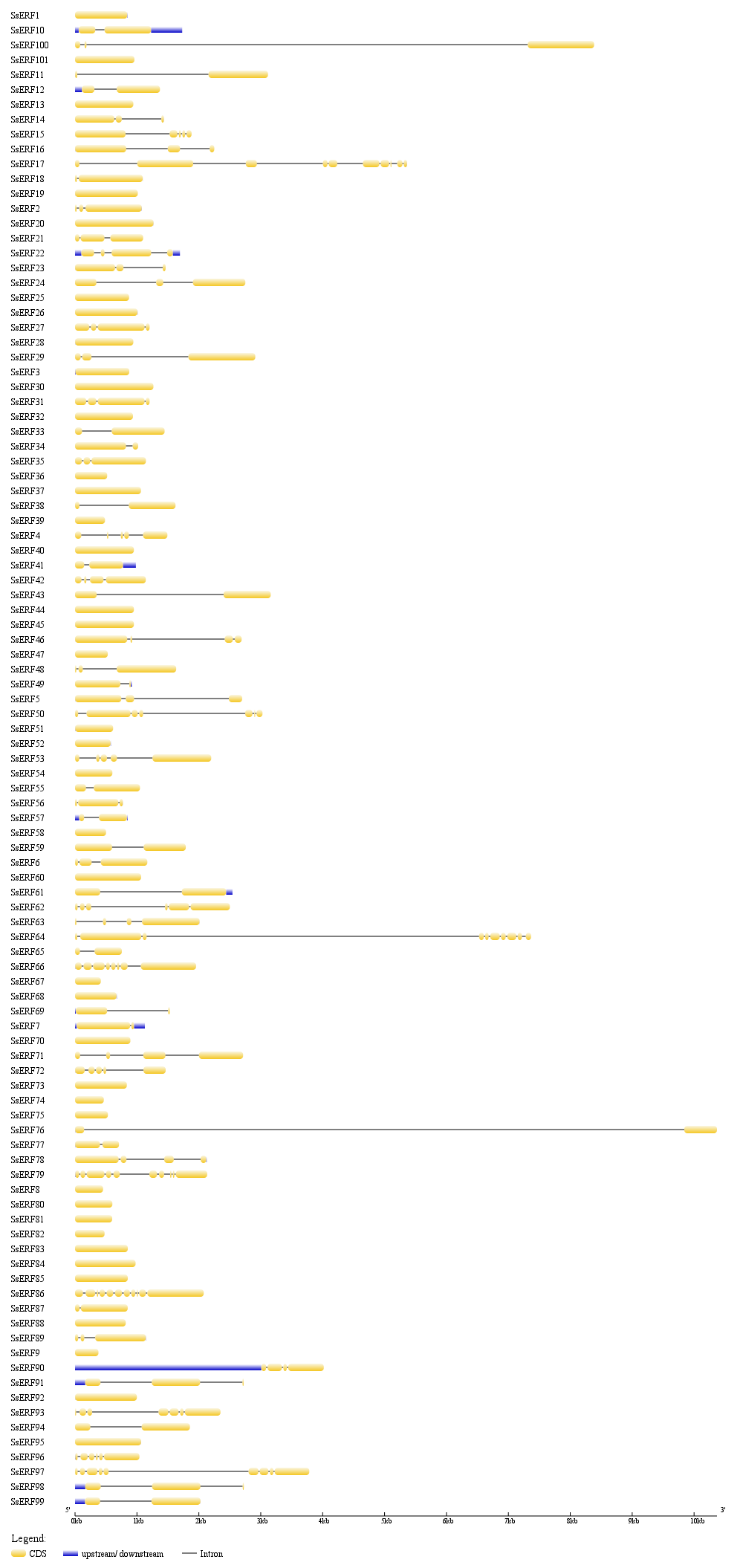


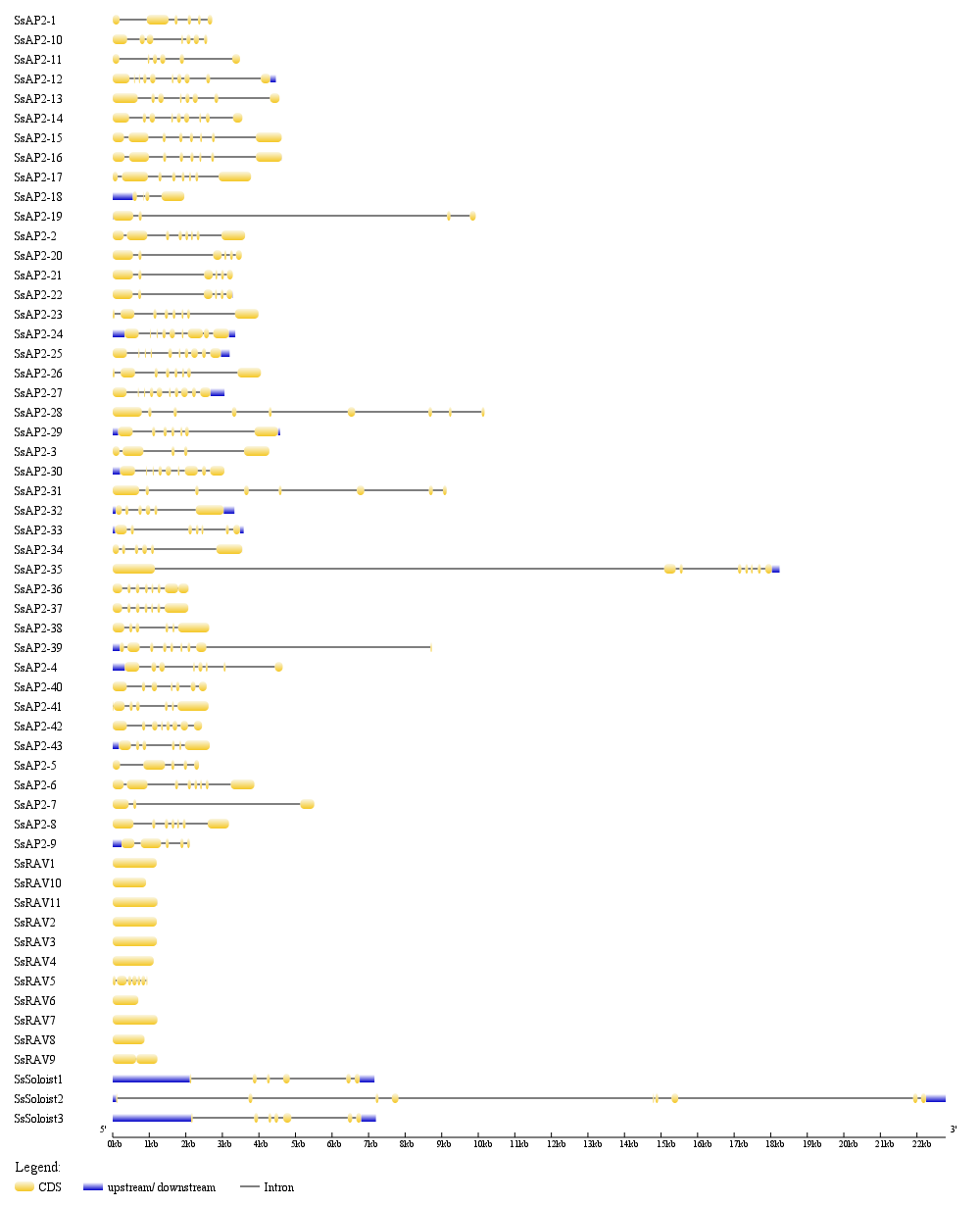


**Fig. S6** Gene structures of *AP2/ERF* superfamily genes. The exon/intron structure was visualized by the Gene Structure Display Server 2.0 (Visualized). The data of gene structure was based on the gene annotation model of *Saccharum spontaneum* L. genome. The yellow box indicates CDS, the gray lines indicate introns, and the blue box indicates untranslated 5-and 3 –regions.
