# Supplementary material for: Genome-wide identification and expression analysis of AP2/ERF transcription factors in sugarcane (Saccharum spontaneum L.)
Source: BMC Genomics. 2020 Oct 2;21:685. doi: 10.1186/s12864-020-07076-x (PMC7531145; doi:10.1186/s12864-020-07076-x)
Supplement: Supplementary file 12 — Additional file 12. Tissue-specific expression profile of AP2 / ERF gene in sugarcane. [file 12864_2020_7076_MOESM12_ESM.docx]

**Additional file 12** Tissue specific expression profile of AP2 / ERF gene in Sugarcane


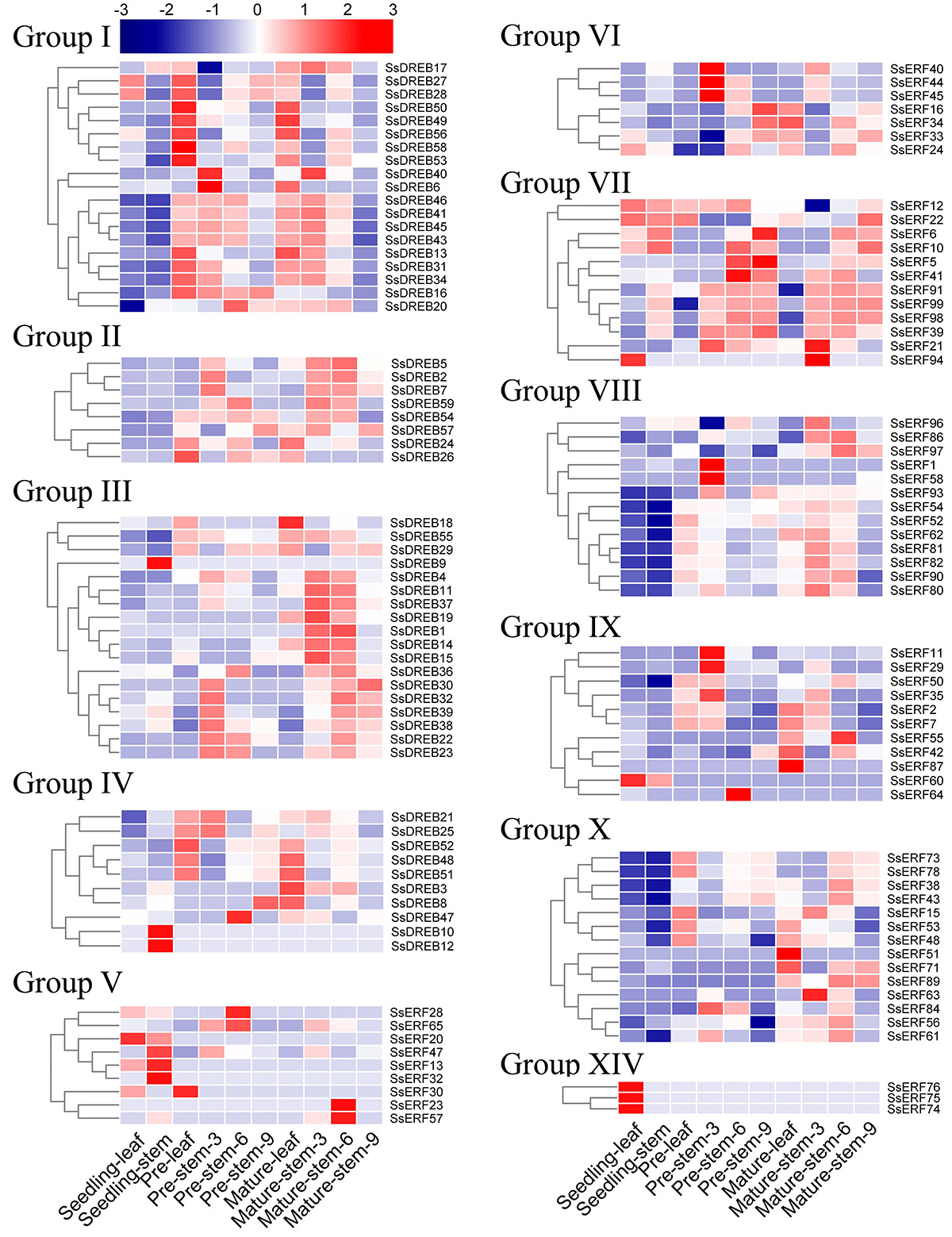


**Fig. S7 Expression profile of sugarcane *ERF* and *DREB* genes, subdivided into groups (I-XIV).** Heat map hierarchical clusters of SsERF and SsDREB genes expression profiles in leaves and stems at the seedling stage, leaves and stems at the early maturity stage (3, 6, 9), leaves, and stems at the mature stage (3, 6, 9). The heatmaps were created by TBtools based on the transformed data of log2 (FPKM+1) values, clusters were generated using the Pearson clustering algorithm. For each line, after z-score-normalized transformation, the expression patterns are presented as heat maps in blue/white/red/ coding, red indicating high expression level, white indicating middle expression level, and blue indicating low expression level.

**
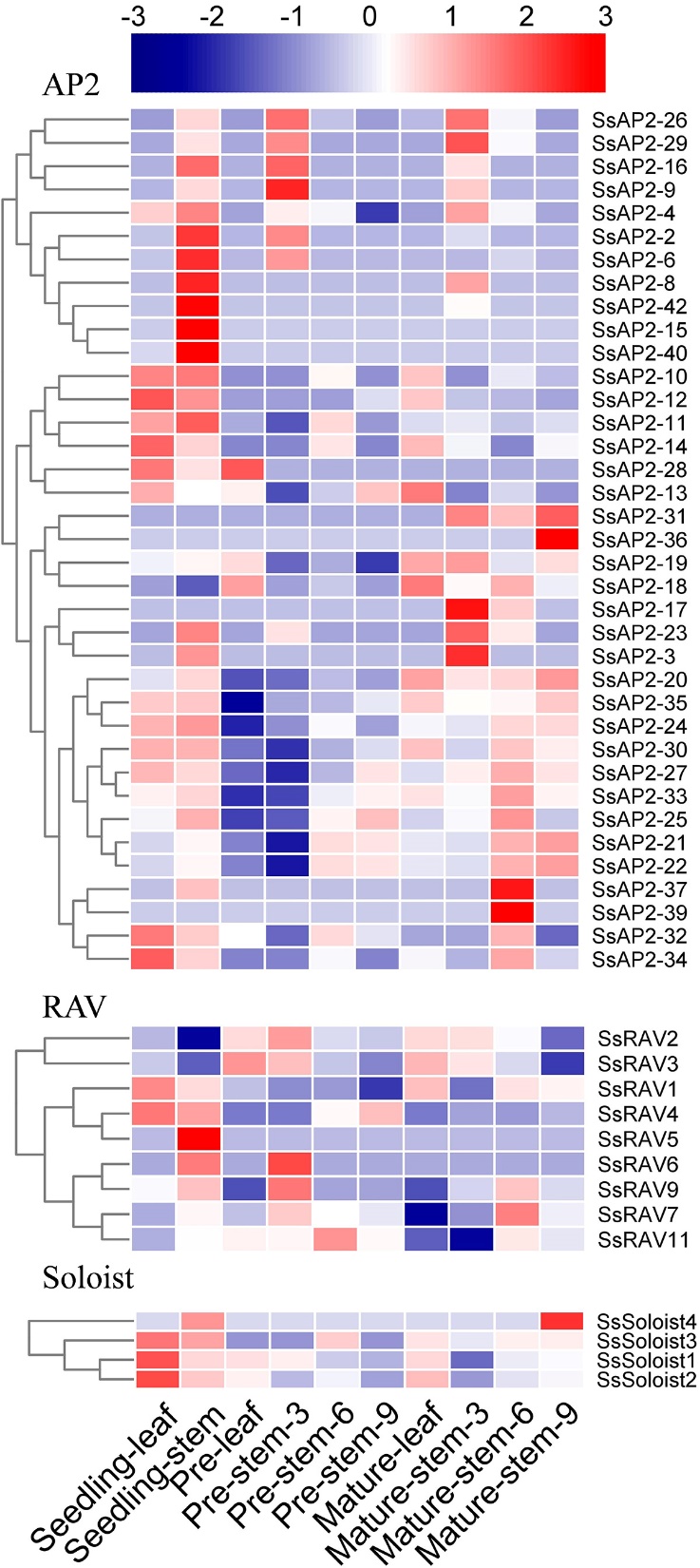
**

**Fig. S8** **Expression profiles of the sugarcane *AP2*, *RAV*, *Soloist* genes**. Thermal map hierarchical clusters of *SsAP2*, *SsRAV*, *SsSoloist* genes expression patterns in leaves and stems at the seedling stage, leaves and stems at the early maturity stage (3, 6, 9), leaves and stems at the mature stage (3, 6, 9). The heatmaps were created by TBtools based on the transformed data of log2 (FPKM+1) values, clusters were generated using the Pearson clustering algorithm. For each line, after z-score-normalized transformation, the expression patterns are presented as heat maps in blue/white/red/ coding, red indicating high expression level, white indicating middle expression level, and blue indicating low expression level.
