# Supplementary material for: Genome-wide identification and expression analysis of AP2/ERF transcription factors in sugarcane (Saccharum spontaneum L.)
Source: BMC Genomics. 2020 Oct 2;21:685. doi: 10.1186/s12864-020-07076-x (PMC7531145; doi:10.1186/s12864-020-07076-x)
Supplement: Supplementary file 13 — Additional file 13 Expression profiles of 12 selected SsAP2/ERF genes in response to various abiotic stress treatments and hormone treatments. [file 12864_2020_7076_MOESM13_ESM.docx]

**Additional file 13** Expression profiles of 12 selected *SsAP2/ERF* genes in response to various various abiotic stress treatments and hormone treatments.


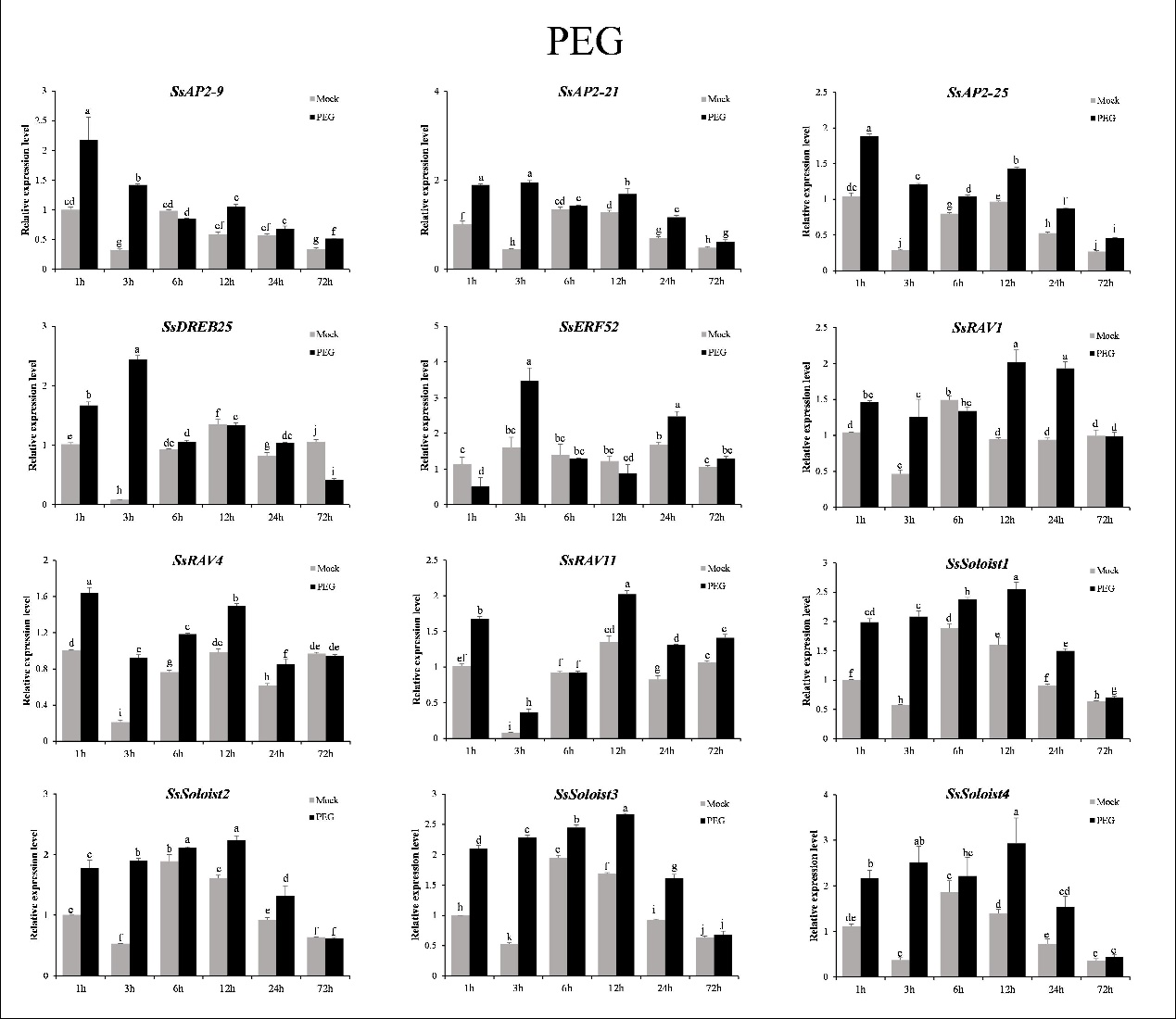


**Fig. S9 Expression profiles of 12 selected SsAP2/ERF genes in response to dehydration stress treatments.** 25s-RNA gene was used as the internal control and to normalize expression data. Relative transcript abundance is normalized relative to S mock (1 hour untreated control group) treatment. Error bars represent the standard deviation of the mean. For each gene, different lowercase letters indicate significant differences among mean values (one-way ANOVA with Ducan’s multiple range test; P < 0.05). The results are based on three replicates in three independent experiments.


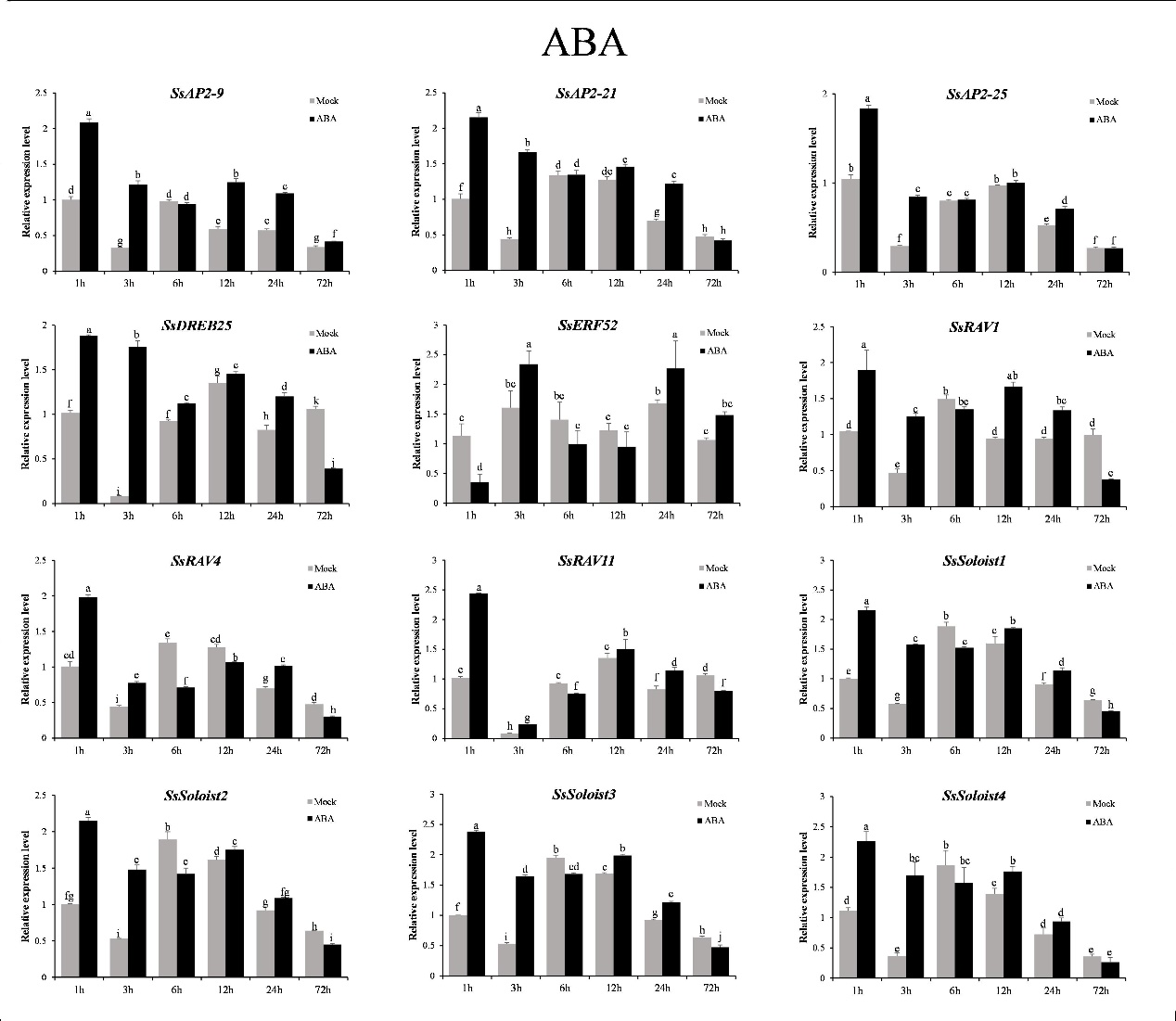


**Fig. S10 Expression profiles of 12 selected SsAP2/ERF genes in response to ABA treatments.**


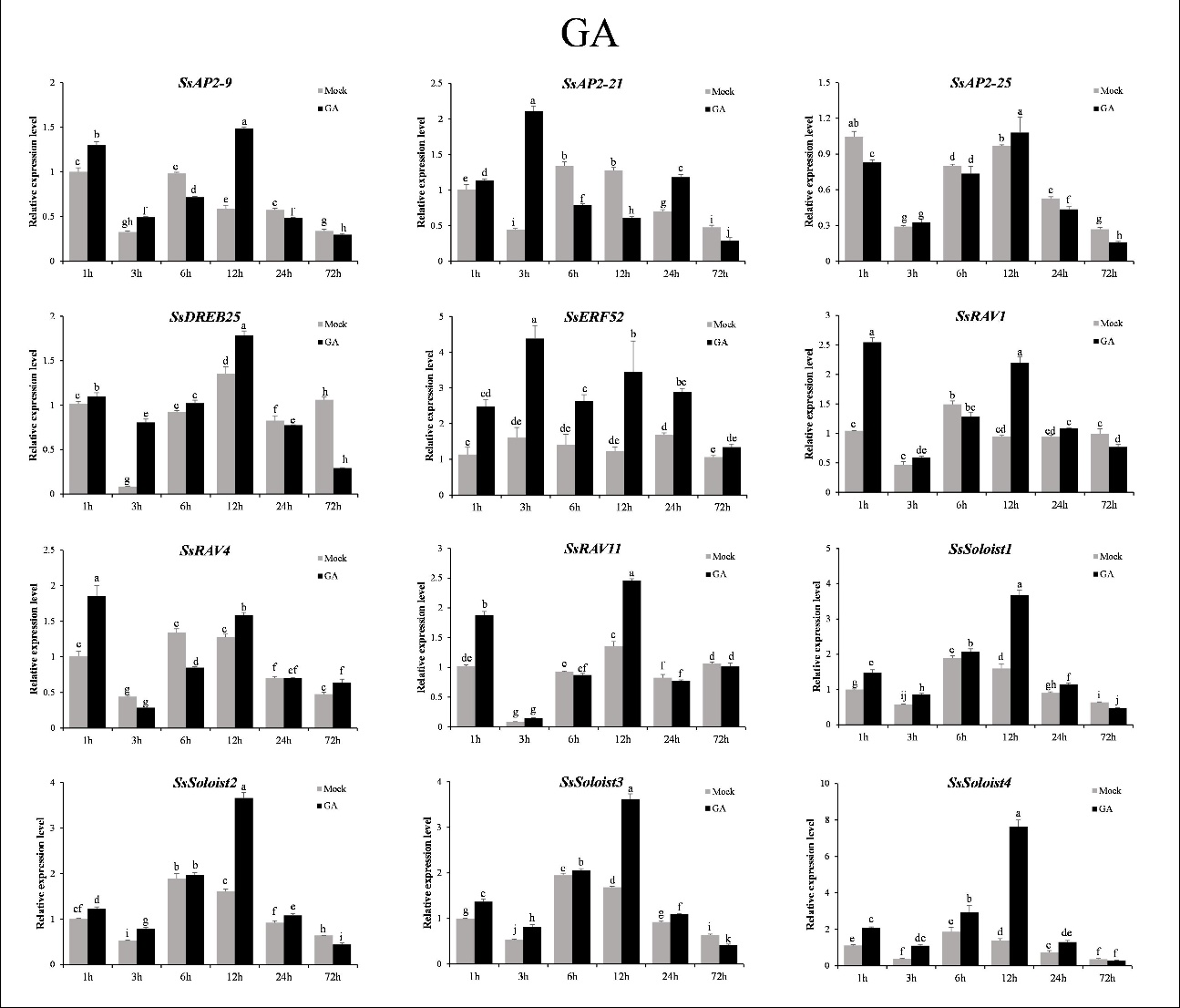


**Fig. S10 Expression profiles of 12 selected SsAP2/ERF genes in response to GA treatments.**
